# Management of Aneurysmal Subarachnoid Hemorrhage During Pregnancy with a Devastating Clinical Course: A Case Report

**DOI:** 10.3390/jcm15072718

**Published:** 2026-04-03

**Authors:** You Sub Kim, Sung Pil Joo, Tae Sun Kim

**Affiliations:** Department of Neurosurgery, Chonnam National University Hospital and Medical School, Gwangju 61469, Republic of Korea; nsjsp@chonnam.ac.kr (S.P.J.); taesun1963@yahoo.co.kr (T.S.K.)

**Keywords:** subarachnoid hemorrhage, pregnancy, clipping, vasospasm, endovascular treatment, shunt

## Abstract

**Background:** Aneurysmal subarachnoid hemorrhage (SAH) during pregnancy is rare, occurring in approximately 0.01–0.05% of pregnancies, most commonly in the third trimester. Its management is particularly challenging, requiring careful consideration of both maternal and fetal outcomes. **Methods:** We report the case of a 32-year-old woman at 31 weeks of gestation who presented with severe headache and left third cranial nerve palsy. Imaging revealed diffuse SAH with significant obstructive hydrocephalus and a 5 mm left posterior communicating artery aneurysm. Following multidisciplinary discussion, surgical clipping was performed while preserving the pregnancy to allow for fetal lung maturation. On postoperative day 8, the patient developed right-sided weakness and aphasia secondary to severe vasospasm. Initial management with catecholamine-induced hypertension resulted in increased uterine contractions and fetal distress. Subsequent intra-arterial administration of nimodipine effectively resolved the vasospasm, enabling cessation of vasopressor therapy. After achieving fetal lung maturity, cesarean section was performed at 34 weeks, followed by ventriculo-peritoneal shunt placement for communicating hydrocephalus. Due to sustained shunt failure, the distal catheter was finally inserted into the superior vena cava at the junction of the atrium. **Results:** The patient showed gradual neurological recovery with complete resolution of third cranial nerve palsy, and both mother and infant were discharged without complications. **Conclusions:** This case highlights that while standard vasospasm therapies can be implemented during pregnancy, hemodynamic approaches may provoke maternal and fetal complications. Endovascular rescue strategies should be promptly considered for severe vasospasm, and ventriculo-atrial shunting for complex communicating hydrocephalus may serve as a viable alternative option in post-cesarean patients.

## 1. Introduction

The incidence of aneurysmal subarachnoid hemorrhage (SAH) during pregnancy is rare, occurring in approximately 0.01–0.05% of all pregnancies, with most cases reported during the third trimester or the puerperium period. It represents the third leading cause of maternal mortality, accounting for 5–12% of maternal deaths [1,2,3]. Physiological changes associated with pregnancy—including increased blood volume, elevated cardiac output, and hormonal influences on vascular integrity—may contribute to the formation or rupture of cerebral aneurysms [4,5].

Despite advances in multidisciplinary treatment strategies, the management of aneurysmal SAH during pregnancy remains complex, as it requires the concurrent consideration of both maternal and fetal outcomes. This creates a clinical dilemma involving diagnostic procedures, anesthetic considerations, the timing and mode of delivery, and the management of the aneurysm itself. Optimal treatment decisions must be made through close collaboration among neurosurgeons, neurointerventionalists, obstetricians, anesthesiologists, and neonatologists, in order to address the intricate balance between neurosurgical and obstetric priorities and to minimize risks to both mother and fetus. Previous case series have reported various aspects of care, including the timing and mode of delivery and strategies for aneurysm treatment [6,7,8]. While some degree of consensus has been reached in these areas, few reports have specifically focused on the management of complications arising during the clinical course of SAH—such as vasospasm and hydrocephalus—particularly in scenarios where pregnancy is maintained after aneurysm treatment.

The aim of the present case is to describe the devastating clinical course of aneurysmal SAH in a pregnant patient, with specific emphasis on the approach to complications secondary to SAH

## 2. Case Presentation

**Figure 1:**
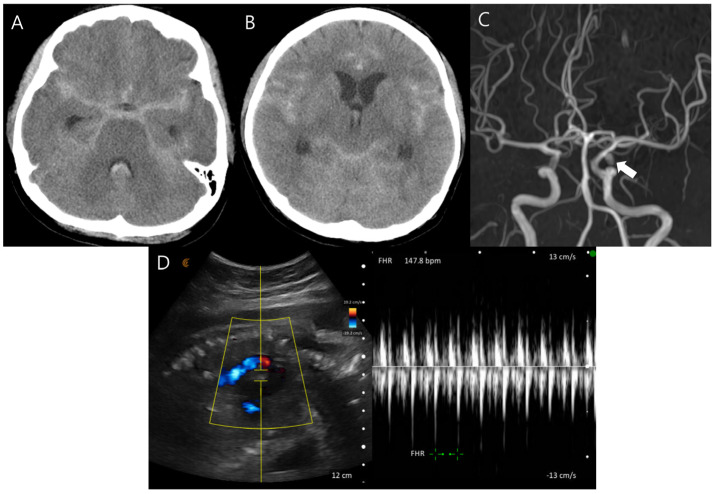
(**A**,**B**) Initial computed tomography (CT) shows diffuse subarachnoid hemorrhage with significant obstructive hydrocephalus. (**C**) Magnetic resonance angiography demonstrates a 5 mm-sized aneurysm at the left posterior communicating artery (white arrow). (**D**) Obstetric ultrasonography shows a stable fetal heart rate. The yellow area represents the sample region used for Doppler flow analysis.

A 32-year-old woman at 31 weeks of gestation presented to the emergency department with a sustained severe headache and left third cranial nerve palsy (Hunt and Hess grade II). Computed tomography (CT) revealed diffuse SAH extending into the third and fourth ventricles (Fisher grade IV), accompanied by marked obstructive hydrocephalus (Figure 1A,B). Magnetic resonance (MR) angiography identified a 5 mm aneurysm at the origin of the left posterior communicating artery (Figure 1C). MR-based imaging was initially attempted; however, prolonged positioning was difficult due to the patient’s initial unstable condition and advanced pregnancy (third trimester). Therefore, hemorrhage was first confirmed by CT, and MR angiography was subsequently performed after the patient became stabilized.

**Figure 2:**
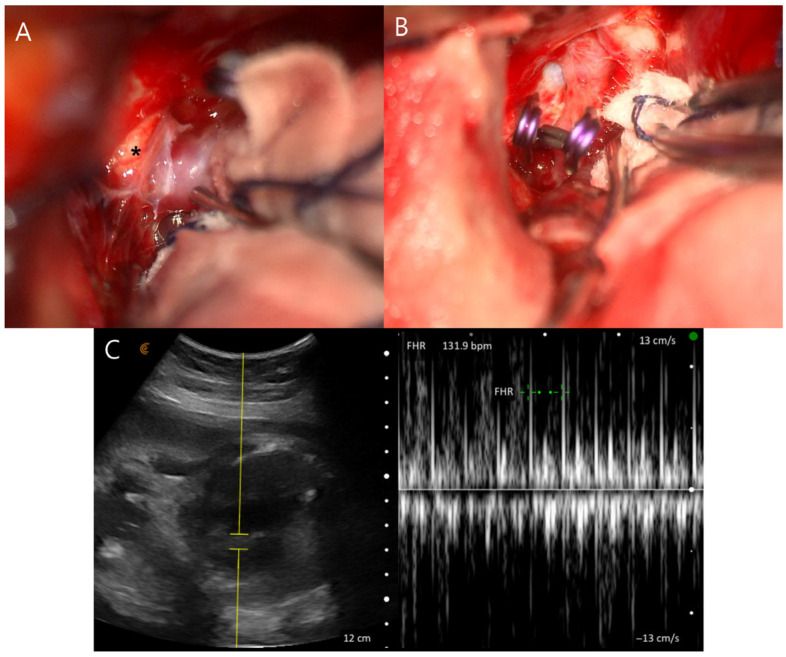
Intraoperative findings. (**A**) Following left frontotemporal craniotomy and Sylvian dissection, the aneurysm at the left posterior communicating artery was confirmed, with compression of the third cranial nerve (asterisk). (**B**) The aneurysm was completely clipped. (**C**) Postoperative ultrasonography confirmed fetal stability. The yellow area represents the sample region used for Doppler flow analysis.

The patient’s neurological status and vital signs, including blood pressure, remained stable under low-dose nicardipine infusion, and fetal well-being was also confirmed (Figure 1D). After multidisciplinary discussion involving neurosurgery, neuroradiology, anesthesiology, obstetrics, and neonatology, it was decided to proceed with aneurysm treatment while maintaining the pregnancy to allow for fetal lung maturation. Given the presence of significant hydrocephalus and the potential risks associated with endovascular intervention during pregnancy—including incomplete aneurysm occlusion, antiplatelet use, and fetal exposure to radiation and contrast media—surgical clipping was performed with ventricular catheter placement under general anesthesia with induction using propofol and maintenance with sevoflurane and remifentanil (Figure 2A,B). Both maternal and fetal conditions remained stable postoperatively (Figure 2C). The patient was transferred to the intensive care unit, and the fetus was monitored with Doppler ultrasonography every 4 h. On postoperative day 7, the ventricular catheter was replaced with continuous lumbar drainage to reduce the risk of catheter-related infection.

**Figure 3:**
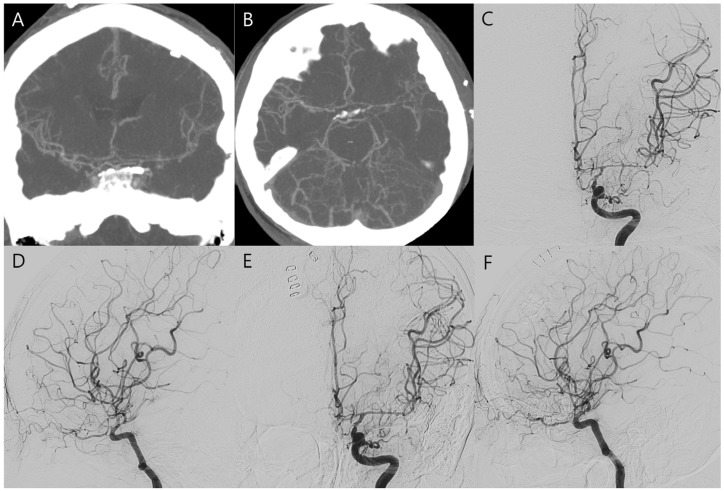
(**A**–**D**) On postoperative day 8, CT angiography and subsequent digital subtraction angiography demonstrate severe vasospasm, especially in the left internal carotid artery, left anterior cerebral artery, and left middle cerebral artery. Following intra-arterial nimodipine infusion, the vasospasm improved (**E**,**F**).

**Figure 4:**
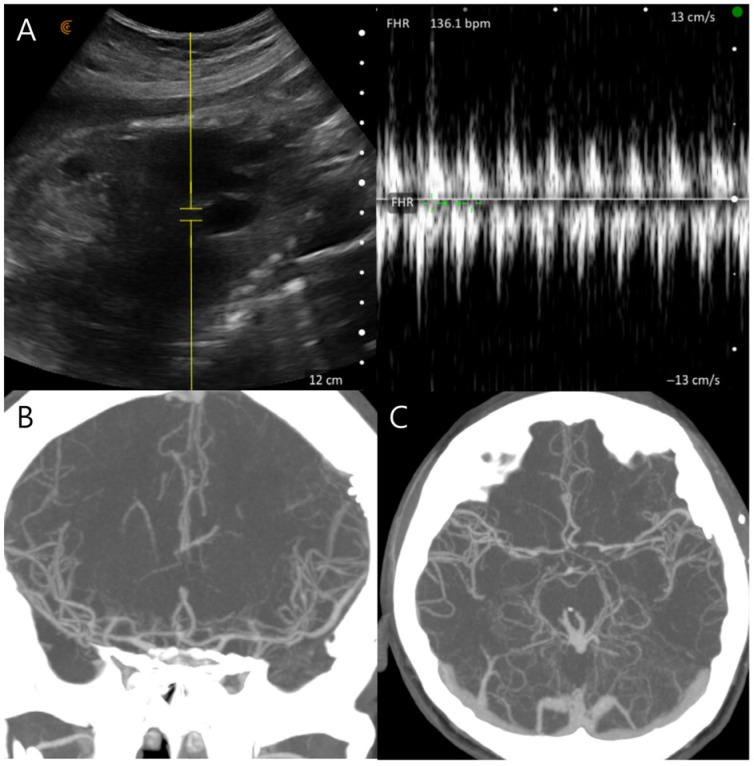
(**A**) The fetus remained reactive after chemical angioplasty. The yellow area represents the sample region used for Doppler flow analysis. (**B**,**C**) Follow-up CT angiography, performed 2 days after chemical angioplasty, reveals further improvement of the vasospasm.

**Figure 5:**
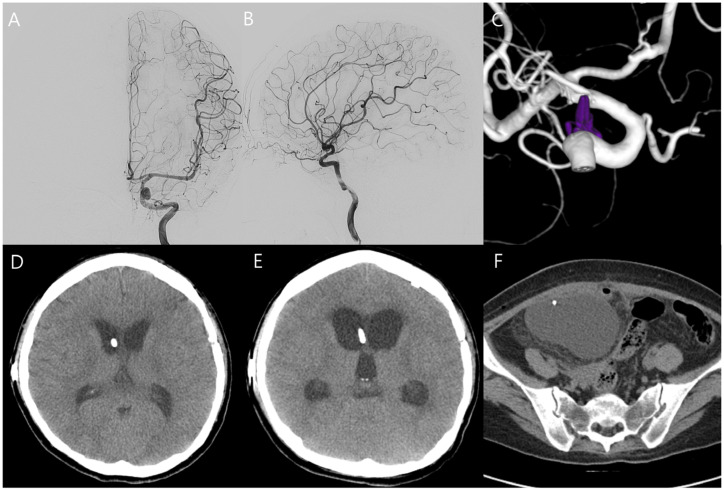
(**A**,**B**) Follow-up angiography after delivery reveals resolution of vasospasm and (**C**) 3D rotational angography demonstrates complete occlusion of the aneurysm. (**D**) Follow-up brain and abdomen CT at 11 days after ventriculo-peritoneal shunt, reveal recurrent hydrocephalus due to shunt failure with pseudocyst formation in abdominal cavity (**E**,**F**).

**Figure 6:**
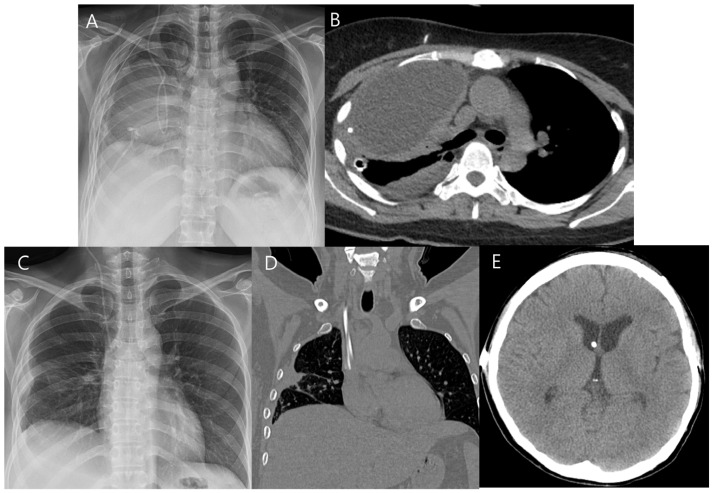
(**A**,**B**) Following the distal catheter was repositioned into the pleural cavity at 3 weeks after ventriculo-peritoneal shunt, large amount of pleural effusion was developed. (**C**–**E**) Distal catheter tip was repositioned into the right superior vena cava at the junction of the atrium (ventriculo-atrial shunt) and sustained improvement of hydrocephalus was achieved.

On postoperative day 8, the patient experienced sudden-onset right-sided weakness and moderate aphasia. CT angiography revealed severe cerebral vasospasm, most prominent in the left internal carotid and middle cerebral arteries (Figure 3A,B). Initial management included induced hypertension using low-dose catecholamines initiated at 0.1 μg/kg/min under intensive maternal-fetal monitoring. Although the patient showed transient improvement, catecholamine requirements escalated to 0.2 μg/kg/min to maintain target arterial pressure of approximately 20% above baseline, and Doppler monitoring revealed frequent uterine contractions and fetal heart rate decelerations correlated with catecholamine dosage. Urgent intra-arterial nimodipine infusion was performed in the angiographic suite with a total dose of 3 mg administered over 20 min, resulting in angiographic and clinical improvement of the vasospasm and permitting cessation of catechoamines (Figure 3C–F). Fetal movements and heart rate remained stable in serial Doppler assessments and ultrasonography (Figure 4A,B). Sustained clinical and radiologic improvement was confirmed by follow-up CT angiography performed two days after angioplasty (Figure 4C,D). Following achievement of fetal lung maturation and in anticipation of additional surgery for communicating hydrocephalus, a planned cesarean delivery was performed at 34 weeks of gestation. A healthy infant weighing 1960 g was delivered. Subsequent digital subtraction angiography confirmed complete occlusion of the aneurysm and resolution of vasospasm (Figure 5A–C). To address sustained hydrocephalus, a ventriculo-peritoneal shunt was placed at 3 weeks after delivery, with follow-up CT showing marked reduction in ventricular size at 7 days after shunt (Figure 5D). However, the patient developed worsening drowsiness with radiologic evidence of recurrent hydrocephalus at 11 days after shunt (Figure 5E). Despite all treatment efforts during another 10 days—including pressure adjustment, repeated flushing of the shunt device, and direct inspection of the distal catheter tip by revision surgery—hydrocephalus persisted and pseudocyst was newly detected in abdomen CT (Figure 5F). As a second option, the distal catheter was repositioned into the right pleural cavity to create a ventriculo-pleural shunt at 3 weeks after ventriculo-peritoneal shunt. However, large amount of pleural effusion was developed and sustained with chest pain and dyspnea (Figure 6A,B). Ultimately, ventriculo-atrial shunt was performed. The distal tip was repositioned into the super vena cava at the junction of the right atrium, and follow-up brain CT showed improvement of hydrocephalus (Figure 6C–E). Gradual neurological recovery ensued, with complete resolution of the third cranial nerve palsy. Both the mother and infant were discharged in stable condition without complications.

## 3. Discussion

The primary therapeutic goal in managing aneurysmal SAH during pregnancy is to achieve complete aneurysmal occlusion while ensuring an uncomplicated delivery. Optimal decision-making requires close interdisciplinary collaboration between neurosurgical and obstetrical teams and should be individualized taking into account gestational age, fetal status, and maternal clinical condition. Based on the established consensus that ruptured aneurysms in pregnancy should be treated as promptly as in non-pregnant patients, the mode and timing of delivery is the remaining task [9,10].

In general, the mode of delivery does not appear to be significantly associated with gestational trimester and does not influence maternal or fetal outcomes in the setting of aneurysmal SAH during pregnancy. Although vaginal delivery can theoretically be performed with adequate analgesia and by avoiding Valsalva maneuvers to minimize hemodynamic fluctuations, this option is often limited to term or near-term pregnancies and is typically feasible only when there is a sufficient interval between aneurysm treatment and delivery [1,11]. In this context, cesarean section (CS) has been widely employed and considered the preferred mode of delivery. CS may offer benefits to both the mother and fetus by reducing the risk of fetal distress during labor, facilitating subsequent management of aneurysmal complications as well as simplifying surgical planning. Furthermore, if labor is expected to occur soon after aneurysm treatment or if aneurysm occlusion remains incomplete, CS may represent a more feasible and safer delivery option. Two large case series have reported that over 70% of pregnant women with aneurysmal SAH underwent CS [7,8].

The timing of delivery in cases of aneurysmal SAH during pregnancy is highly variable. In a pooled analysis of published case series, Robba et al. reported that delivery occurred at full term in 14.3% of cases, after aneurysm treatment in 30.6%, before aneurysm treatment in 24.5%, and concurrently with aneurysm treatment in 22.4% [8]. In that analysis, the majority of cases (77.5%) favored early delivery immediately before, during, or after aneurysm treatment, likely reflecting the fact that over 70% of SAH events occurred in the third trimester, when fetal maturation is typically sufficient to permit delivery. Nevertheless, once the aneurysm has been secured, immediate delivery is not always required at the time of SAH diagnosis. In cases where maternal and fetal status remain stable, pregnancy may be safely continued, if needed. In the present case, given the stability of both maternal and fetal conditions following aneurysm treatment, we decided to maintain the pregnancy beyond 34 gestational weeks to allow for fetal lung maturation. A planned CS was subsequently performed after reaching this gestational threshold.

Another important consideration in the management of aneurysmal SAH during pregnancy is the choice between surgical clipping and endovascular treatment. Although endovascular treatment is less invasive, concerns regarding fetal radiation exposure, contrast use, and the need for antiplatelet therapy limit their applicability in selected cases [12,13]. The angiography suite also presents challenges for continuous fetal monitoring and does not readily accommodate emergent delivery scenarios. In contrast, surgical clipping allows definitive aneurysm occlusion and facilitates intraoperative fetal monitoring, making it a suitable option when pregnancy is to be maintained particularly for aneurysms with morphologies such as small, broad-neck lesions that are less amenable to endovascular treatment [14]. In the present case, surgical clipping was performed to avoid the risks associated with endovascular treatment—specifically, incomplete occlusion and the requirement for dual antiplatelet therapy—given our intention to maintain the pregnancy beyond 34 weeks’ gestation.

Cerebral vasospasm associated with aneurysmal SAH during pregnancy may present more severely than in non-pregnant patients. Although evidence remains limited, hormonal and physiological changes inherent to pregnancy likely contribute to the severity of vasospasm [15,16]. Altered hemodynamics, a hypercoagulable state, and the inflammatory milieu resulting from blood breakdown products may act synergistically to exacerbate cerebral vasospasm. In addition, hormonal influences may alter vascular reactivity, affecting the brain’s susceptibility to vasospasm.

Hemodynamic augmentation is commonly used as an initial strategy for managing cerebral vasospasm in non-pregnant patients and is also applied in pregnant patients, but its application requires careful adjustment of target parameters [17]. Induced hypertension is typically achieved by increasing mean arterial pressure (MAP) by 20–30% above baseline or targeting a systolic blood pressure of 160–200 mmHg. However, as in our case, although initial low-dose catecholamine administration resulted in transient neurological improvement, escalating vasopressor requirements led to frequent uterine contractions and fetal heart rate decelerations, highlighting the narrow therapeutic window of hemodynamic augmentation in this setting. In cases of medically refractory vasospasm, endovascular rescue interventions such as intra-arterial vasodilator infusion and balloon angioplasty can be employed, similar to their use in non-pregnant populations [17]. The cornerstone of effective management in this context is continuous maternal-fetal monitoring during hemodynamic therapy, which enables timely detection of uterine contractions, cardiopulmonary compromise, and fetal distress. This vigilance facilitates prompt decision of rescue endovascular procedures, as was necessary in our case. Delay in such interventions may result in excessive fluid administration and vasopressor use, potentially leading to maternal and fetal morbidity or mortality. In our case, CT angiography was performed prior to delivery to evaluate severe vasospasm. Although MR angiography was initially considered, it was technically limited due to patient positioning difficulties in the third trimester and motion artifacts. Therefore, CTA was performed as a rapid and reliable modality following multidisciplinary discussion, balancing maternal neurological risk against potential fetal exposure. Importantly, the fetal radiation dose from maternal head CT is considered negligible, as the uterus lies outside the primary beam and appropriate shielding is applied [18]. Moreover, iodinated contrast agents have not been shown to be associated with teratogenic effects in the third trimester when organogenesis is largely complete and the susceptibility to teratogenic exposure is reduced although their use during pregnancy should be carefully justified [19]. Current guidelines emphasize that clinically indicated diagnostic imaging should not be delayed during pregnancy, as postponement may increase maternal risk and potentially lead to adverse fetal outcomes, including fetal distress [20].

The management of communicating hydrocephalus in our case was complicated by repeated shunt failure following CS. Although ventriculo-peritoneal shunting remains the standard treatment, physiological and anatomical changes associated with pregnancy and the postpartum state may contribute to distal catheter dysfunction [21]. In cases of refractory shunt failure, alternative approaches such as ventriculo-atrial shunting may be considered as a salvage option.

## 4. Conclusions

The management of aneurysmal subarachnoid hemorrhage during pregnancy requires individualized decision-making regarding the timing and mode of delivery, as well as the method of aneurysm treatment. A multidisciplinary approach is essential to optimize outcomes for both the mother and fetus. When pregnancy is to be continued following aneurysm treatment, careful monitoring is imperative, with proactive management of SAH-related complications such as vasospasm and hydrocephalus. In cases of severe vasospasm, treatment should mirror that of non-pregnant patients; however, heightened monitoring is necessary, and prompt endovascular rescue procedures should be prioritized, as hemodynamic therapy may pose risks to both the mother and fetus. For complex hydrocephalus in previous CS patient, a ventriculo-atrial shunt may serve as a viable alternative in instances of ventriculo-peritoneal or -pleural shunt failure.

## Data Availability

The data that support the findings of this study are available from the corresponding author upon reasonable request.

## Abbreviations

The following abbreviations are used in this manuscript:

SAH	Subarachnoid hemorrhage
CT	Computed tomography
CS	Cesarean section
